# Smartphone-based behaviour analysis for challenging behaviour in intellectual and developmental disabilities and autism spectrum disorder – Study protocol for the ProVIA trial

**DOI:** 10.3389/fnins.2022.984618

**Published:** 2022-10-13

**Authors:** Julia Geissler, Hanna Buchholz, Rinat Meerson, Klaus Kammerer, Manuel Göster, Johannes Schobel, Christoph Ratz, Regina Taurines, Rüdiger Pryss, Marcel Romanos

**Affiliations:** ^1^Department of Child and Adolescent Psychiatry, Psychosomatics and Psychotherapy, Center of Mental Health, University Hospital Würzburg, Würzburg, Germany; ^2^Institute for Clinical Epidemiology and Biometry (IKEB), University of Würzburg, Würzburg, Germany; ^3^DigiHealth Institute, Neu-Ulm University of Applied Sciences, Neu-Ulm, Germany; ^4^Education for People with Developmental and Intellectual Disabilities, University of Würzburg, Würzburg, Germany; ^5^German Centre of Prevention Research in Mental Health, University Hospital Würzburg, Würzburg, Germany

**Keywords:** autism spectrum disorder, intellectual disability, challenging behaviour, behaviour analysis, mental health application, cognitive behaviour therapy, parent training, parental stress

## Abstract

**Background:**

Challenging behaviour (CB) comprises various forms of aggressive and problematic behaviours frequently occurring in children with intellectual and developmental disability (IDD) or autism spectrum disorder (ASD). CB often arises from impaired communication or problem solving skills. It is often met with coercive measure due to a lack of alternative strategies on the part of the caregiver, while it also impacts on the caregivers due to the exposure to physical harm and high levels of stress. Within the ProVIA project we developed a smartphone-based tool for caregivers of children with IDD and/or ASD to prevent and modify CB. The ProVIA app systematically helps caregivers to identify specific causes of CB and provides individualised practical guidance to prevent CB and consecutive coercive measures, thus aiming to improve the health and well-being of the children and caregivers.

**Methods:**

In this uncontrolled open trial we will enrol *N* = 25 caregivers of children aged 3–11 years with a diagnosis of IDD and/or ASD. Participants will use the ProVIA-Kids app for 8 weeks. During the intervention phase, participants will conduct behaviour analyses after each instance of CB. The app will summarise the identified putative causes for the CB in each situation, and provide recommendations regarding the handling and prevention of CB. Furthermore, the app will aggregate data from all available behaviour analyses and identify the most relevant (i.e., most frequently reported) risk factors. Measurement points are at baseline (T0), after the intervention (T1) and 12 weeks after the end of the intervention (follow-up; T2). The primary outcome is the absolute change in parental stress (EBI total scale) between T0 and T1. Further aspects of interest are changes in CB severity and frequency, caregiver mood, satisfaction with the parenting role (EFB-K total scale) and experienced parenting competence (FKE total scale). Pre-post comparisons will be analysed with paired sample *t*-tests.

**Discussion:**

ProVIA is pioneering structured behaviour analysis via smartphone, assessing predefined causes of CB and providing feedback and recommendations. If this approach proves successful, the ProVIA-Kids app will be a valuable tool for caregivers to prevent CB and improve their own as well as the children’s quality of life.

**Trial registration:**

The study is registered at https://www.drks.de/drks_web/navigate.do?navigationId=trial.HTML&TRIAL_IDDRKS00029039 (registered May 31, 2022).

## Introduction

Approximately 1% of the population has an intellectual and developmental disability (IDD; IQ < 70). Children and adolescents with IDD represent a high-risk clientele for somatic and psychiatric disorders that interact, cause complex multiple disabilities, and result in significant challenges for medical, psychotherapeutic, and educational care (Häßler et al., 2014). Especially autism spectrum disorder (ASD) is highly comorbid with IDD. Among children with ASD, approximately 45% also have a diagnosis of IDD. (Saemundsen et al., 2013). ASD has a overall prevalence of 0.9–1.1% (AWMF, 2016) and core symptoms comprise difficulties in social interaction, verbal and non-verbal communication as well as limited, repetitive and stereotyped patterns of behaviour, interests and activities. Symptoms manifest in early childhood and often confer lifelong impairment (Baxter et al., 2014). Like IDD, ASD is classified as a neurodevelopmental disorder according to the DSM-5 (American Psychiatric Association, 2013). About 52% of children with IDD show challenging behaviour (CB). Common manifestations of CB are e.g., auto-aggression, aggression against others or directed at objects, or pervasive refusal (Kahng et al., 2002; McClintock et al., 2003; Dworschak et al., 2016). The risk for auto-aggression and aggression toward others increases by 31% when IDD is accompanied by comorbid ASD (Tsiouris et al., 2011).

There is significant overlap in the risk factors of CB between children with IDD and children with ASD (NICE, 2013, 2015; Häßler et al., 2014). Risk factors are e.g., the inability to communicate emotional states and needs in general (Mancil, 2006; Dominick et al., 2007; Hartley et al., 2008; Gotham et al., 2013; Greenlee et al., 2016), the presence of ADHD symptoms or sleep disturbances (Chen et al., 2017), comorbid psychiatric symptoms (Hayes et al., 2011; Moss et al., 2018) and environmental influences (e.g., sensory stimuli, cognitive and/or social overload or restrictions of repetitive or stereotyped behaviour) (Reese et al., 2005).

### Effects of CB on caregivers

Caregivers of children with ASD and/or IDD experience high levels of stress, especially due to CB. A recent representative survey in residential facilities for young people with IDD in Bavaria, Germany, showed that CB in children and adolescents with IDD is significantly associated with employee stress and the use of coercive measures (Geissler et al., 2021a). Giovagnoli and colleagues were able to show that parental stress was less related to the severity of ASD core symptoms and more related to the extent of certain challenging behaviours (emotional reactiveness, aggression) (Giovagnoli et al., 2015). Expansive behaviour further contributed to a deterioration of the parent-child relationship (Zaidman-Zait et al., 2018). The extent to which parents are burdened by the child’s behaviour results on the one hand from child variables (e.g., hyperactivity/distractibility, adaptability, and mood), but on the other hand is also influenced by parent variables (e.g., social isolation, quality of relationship with or attachment to the child, depressiveness, limitations on own physical and mental functioning, degree of acceptance of the child’s characteristics, and extent of positive reinforcement experienced) (Baker et al., 2005; Glidden et al., 2006; Paczkowski and Baker, 2008; van der Veek et al., 2009; MacDonald et al., 2010).

### Addressing risk factors for challenging behavior

Interventions based on standardised behaviour analyses are an effective tool for identifying the causes of CB and for modifying the behaviour (Dunlap and Fox, 2011; NICE, 2015). A systematic review by MacDonald and McGill concludes that training professionals in dealing with CB via behaviour analytic techniques (Positive Behaviour Support) can reduce the frequency of CB (MacDonald and McGill, 2013). Parents can also be successfully instructed to use behaviour therapy-based interventions with children with ASD (Diggle et al., 2003; Postorino et al., 2017). Randomised-controlled trials show efficacy of the parent-training program Stepping Stones Triple P (SSTP) for children with IDD in terms of the child’s CB, parental competence and satisfaction and parental relationship (Plant and Sanders, 2007; Tellegen and Sanders, 2013). Ruane and Carr (2019) reported meta-analytic effects for SSTP on child behaviour (researcher observed, *d* = 0.51) and child problems (parent report, *d* = 0.46) as well as on parenting style (*d* = 0.70), parenting satisfaction and self-efficacy (*d* = 0.44), parental adjustment (*d* = 0.27) and parental relationship (*d* = 0.26) (Ruane and Carr, 2019).

A meta-analysis found evidence that behavioural parent training also has positive secondary effects on parents’ psychological well-being (Singer et al., 2007). Conversely, trainings explicitly targeting parental mental health may also have a positive impact on children’s behaviour. In a randomised controlled study by Neece (2014), *N* = 46 parents of children with developmental disorders (aged 2.5–5 years) showing behavioural problems underwent 8 weeks of mindfulness-based stress reduction (MBSR). The authors found that compared to the control group, the MBSR group showed greater improvements in terms of parental stress, depression and life satisfaction. Furthermore, the children, who were not part of the intervention, also improved in terms of attention and hyperactivity problems (Neece, 2014). Lewallen and Neece (2015) conducted a study on a similar cohort (parents of *N* = 24 children with developmental disorders aged 2.5–5 years showing behavioural problems). The authors could show that in addition to improvements in the parent-child relationship (relationship frustration, parenting confidence), the intervention also had an effect on the children’s behaviour in terms of improved self-control (parent and teacher rating) as well as cooperation (teacher rating) (Lewallen and Neece, 2015). Furthermore, cognitive-behavioural interventions for parents of children with developmental disorders including problem solving, stress management, and coping with difficult emotions confer improvements in terms of depressiveness, quality of life, and stress compared to a control group (Singer et al., 1988; Nixon and Singer, 1993; Wong and Poon, 2010; Feinberg et al., 2014). To sum up, research has shown that training parents improves CB in children and contributes to parent’s wellbeing, which in turn has positive effects on the children.

### Scarcity of resources

In the reality of care, however, too few resources are available to parents and caregivers. According to health insurance data, children and adolescents with ASD (0–17 years) are most frequently treated by a specialist in paediatrics and adolescent medicine. However, child and adolescent psychiatry topics are underrepresented in medical degree courses (Warnke, 2015). Therefore, it is unlikely that parents receive adequate training in dealing with CB from those providers. Only a small proportion of patients receive psychotherapeutic treatment (6%) (Bachmann and Hoffmann, 2015). In a German interview study, parents of children with ASD were largely satisfied with therapeutic services for their children, but would like to see more counselling and support services for parents (Jungbauer and Meye, 2008). Hence, there is a need for evidence-based low-threshold interventions for caregivers to reduce dependency on external resources. In this respect, MHAs are a promising option.

### Mental health applications

In their systematic review, Miralles and colleagues provide an overview of evidence-based MHAs (Miralles et al., 2020). The number of mental health apps has increased rapidly in recent years. The majority of apps are developed by private companies and are either not scientifically evaluated or their effectiveness has not been proven (Larsen et al., 2016). The majority of evidence-based apps are aimed at patients with affective disorders, anxiety disorders, substance use disorders, psychosis, trauma and stress-related disorders, suicidal behaviour and non-suicidal self-injury, obsessive-compulsive disorders and regulatory disorders. The only two apps developed for ASD with peer-reviewed publications teach children everyday skills using flashcards [iCanLearn; (Zaffke et al., 2014)] or support them in coping with everyday life [LifePal; (Skillen et al., 2016)]. However, these apps were aimed at ASD patients with a high level of functioning who can use the app independently. ProVIA-Kids was developed for caregivers of children with ASD who are more severely impaired (e.g., non-verbal, with cognitive impairment and with severe behavioural problems) and who are often excluded from trials and overlooked in the development of new interventions. Furthermore, there are no MHAs for caregivers with the aim of improving CB in children. However, there is some evidence regarding the efficacy of self-directed interventions in reducing child problem behaviour (Markie-Dadds and Sanders, 2006; Sanders et al., 2007). In terms of parental outcomes, mental health apps can be an effective tool for the reduction of depressive symptoms in adults as part of a multimodal treatment plan [meta-analysis by Firth et al. (2017); 18 RCTs; *g* = 0.38, 95% CI: 0.24–0.52, *p* < 0.001)] and as standalone intervention [meta-analysis by (Weisel et al., 2019); 6 RCTs; Hedges’ *g* = 0.33, 95% CI 0.10–0.57, *P* = 0.005, NNT = 5.43, *I*^2^ = 59%] mostly via behavioural activation. Hence it is possible for self-directed parent interventions to have an effect on both child behaviour and parental well-being. ProVia-Kids is to the best of our knowledge the first app to offer content for children with ASD and/or IDD, which adresses caregivers in the family instead of the affected persons or mental health providers.

### The ProVIA trial

ProVIA aims to provide caregivers with a low-threshold way to improve their understanding of CB and guide them in the modification of the behaviour via a digital intervention tool. Risk factors for CB are screened via a behaviour analysis algorithm. Based on the information entered by caregivers, the app provides detailed psychoeducational information and appropriate recommendations for action for each situation. In addition, a strong focus is placed on strengthening the caregivers’ resources in order to reduce stress and thereby exert a positive secondary effect on the child’s behaviour. For patient safety, the app directs caregivers to specialised settings for issues that require more extensive care.

To our knowledge, ProVIA-Kids is the first attempt to automate behaviour analysis to the extent that users receive feedback on individual risk factors of CB and are recommended matching interventions.

Since the focus of this study is the evaluation of the general suitability of an app-based intervention for caregivers, we defined parental stress as the primary clinical outcome: we expect to see improvements due to a better understanding of the underlying causes of the CB and increased self-efficacy. Considering the short intervention period of 8 weeks, we don’t expect big changes in the children’s (challenging) behaviour. However, in order to avoid overlooking potential early changes in child behaviour, we included this as a secondary exploratory outcome along with other measures of lower priority.

This report presents version 3 (April 20 2022) of the study protocol.

## Methods and analysis

### Study design and trial flow

**FIGURE 1:**
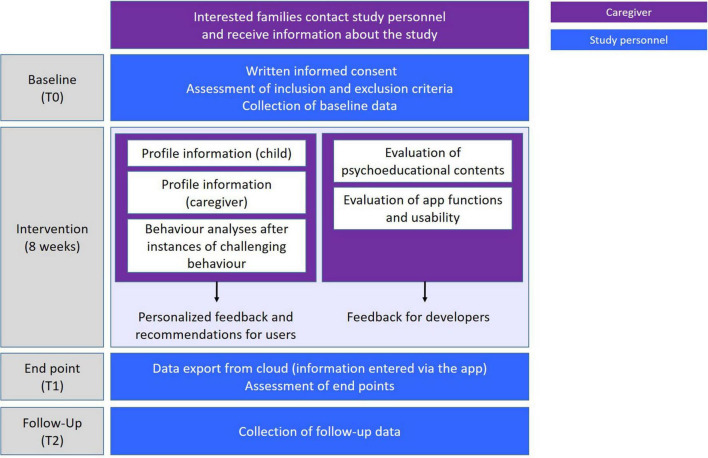
ProVIA trial flow.

Study participants are parents of children with IDD or ASD (“caregivers”). Written informed consent for the trial will be obtained from all persons holding custody of the child by a member of the study team. The study is designed as a pre-post trial without a control group. Since the ProVIA-Kids app is innovative in multiple respects, the goal of this trial is the assessment of app usability and suitability of the recommendations for the target group. Based on the user feedback, the app will be updated and then evaluated in a randomised controlled trial. After the baseline assessment regarding inclusion and exclusion criteria, *N* = 25 caregivers are enrolled in the study. Participating caregivers use the app for 8 weeks. Measurements points are at baseline (T0: screening of inclusion and exclusion criteria, baseline assessment), after the treatment phase (T1) and 12 weeks after the end of the treatment (follow-up; T2). Caregivers who discontinue the intervention are encouraged to participate in the remaining measurement points. For an overview of the trial flow, please refer to Figure 1.

### Participants

The sample comprises caregivers of children aged 3–11 years with a diagnosis of autism spectrum disorder (ASD) and/or Intellectual Developmental Disability (IDD) who display a defined set of challenging behaviours (auto-aggression, aggression directed at others or at objects, pervasive refusal, verbal aggression, excessive vocalisation/screaming). Recruitment will primarily be conducted via the specialised outpatient clinics for ASD and for IDD, the clinic for multiply handicapped children with IDD (Klinik am Greinberg) and the general outpatient clinic at the Department for Child and Adolescent Psychiatry, Psychosomatics and Psychotherapy of the University Hospital Würzburg. Other recruitment strategies may include disseminating information via self-help organisations, paediatricians and other care institutions. Participants will be enrolled if they meet all of the eligibility criteria outlined in Table 1.

**TABLE 1:** Inclusion and exclusion criteria.

**Inclusion criteria**	● Informed consent by caregiver ● Caregiver speaks sufficient German for using the app ● Child’s age: 3;0–11;11 years ● Child diagnosis of IDD (IQ < 70) or/and ASS (diagnosis established by primary care provider)

**Exclusion criteria**	● Caregiver: Severe psychiatric disorder interfering with study participation (parent-report) ● Child: Severe somatic or neurological disorder (parent-report) ● Child: Severe psychiatric comorbidity (parent-report) ● Child: Severe deprivation (parent-report and/or determined by referring physician or therapist) ● Child not living with participating caregiver (e.g., in a residential institution)

### Data handling

The study meets all legal requirements regarding the protection of personal data. Upon enrolment, each participant is assigned a study-specific identification code generated on their smartphone via the app. In order to ensure complete pseudonymisation, all study data collected from participants will be transmitted to the University Hospital of Würzburg (UKW) server and stored under that code. Upon enrolment, participating caregivers share their ID code with the investigators to allow for the paper-based questionnaires to be linked to the data transmitted from the app. Access to the patient identification list is limited to the principal investigators. All data is encrypted before transmission to the UKW to prevent unauthorised access to confidential information. Data quality control measures (e.g., range checks for data values, double checking entered data, plausibility checks) will be performed.

### Intervention

**FIGURE 2:**
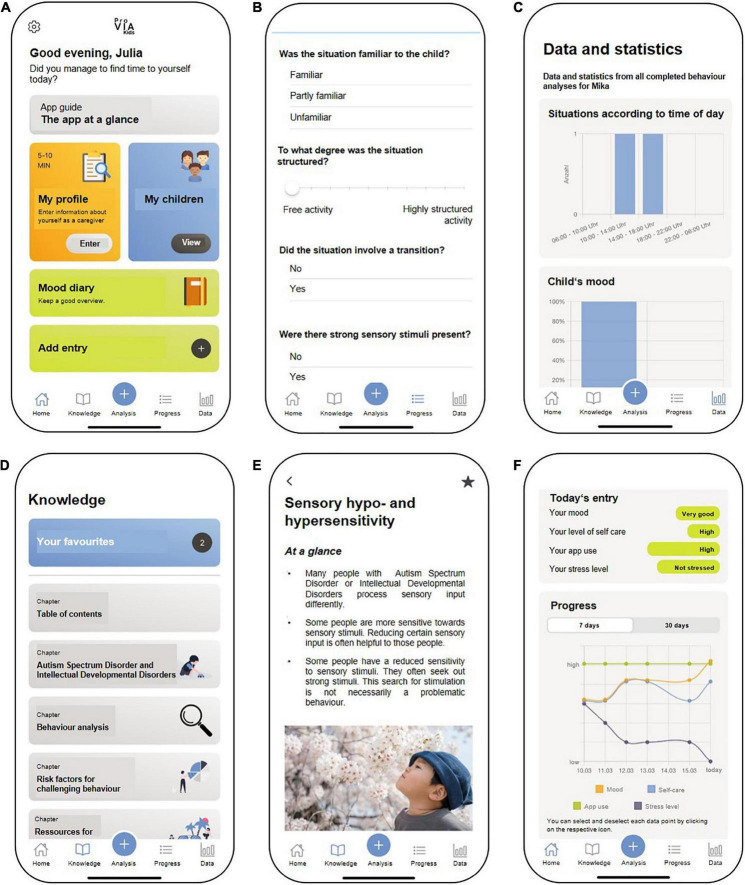
Features of the ProVIA-Kids app (English mock-up, app not yet available in other languages): **(A)** Home screen, **(B)** Behaviour analysis algorithm, **(C)** Graphical display of the frequency of individual contributing factors for CB, **(D)** Menu for psychoeducational content, **(E)** Sample from chapter “Hypersensitivity” with recommendations for caregivers, **(F)** Mood diary. Source: Unsplash: https://unsplash.com/photos/Uu692 wJ0FCY.

**FIGURE 3:**
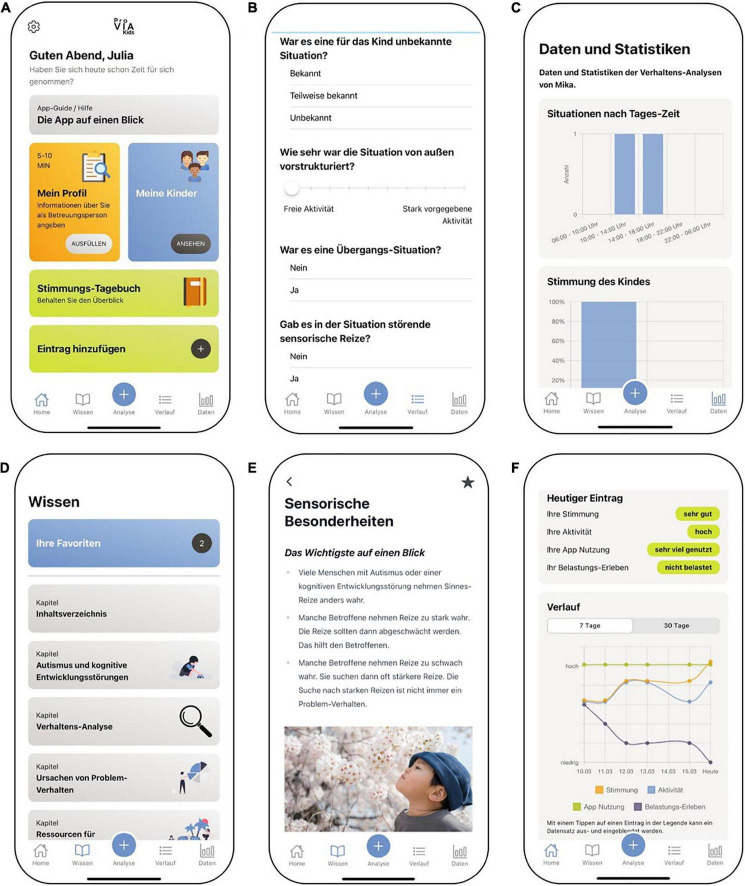
Features of the ProVIA-Kids app (original German version): **(A)** Home screen, **(B)** Behaviour analysis algorithm, **(C)** Graphical display of the frequency of individual contributing factors for CB, **(D)** Menu for psychoeducational content, **(E)** Sample from chapter “Hypersensitivity” with recommendations for caregivers, **(F)** Mood diary.

The ProVIA-Kids app (Figures 2, 3) systematically explores potential risk factors of challenging behaviour, based on which caregivers are provided with appropriate recommendations. The AWMF guidelines on cognitive impairment (Häßler et al., 2014) and autism spectrum disorder (AWMF, 2016, 2021) as well as the NICE guidelines (NICE, 2013, 2015) form the basis for the potential risk factors and the recommendations for action.

#### Compilation of risk factors and recommendations

JG and HB screened the guidelines for mentions of challenging behaviour and the suspected contributing factors and compiled a list of risk factors of CB. This preliminary list was revised and expanded with the clinical expertise from the teams of the specialised outpatient units for ASD and IDD and the clinic for children with multiple disabilities and IDD (Klinik am Greinberg). This list forms the basis of the app’s behaviour analysis algorithm.

The selection of evidence-based recommendations is primarily based on the guidelines for ASD and IDD (NICE, 2013, 2015; Häßler et al., 2014; AWMF, 2021). Psychoeducational information and appropriate strategies for each risk factor were mainly compiled from published treatment manuals and from clinical experience with those patient groups. Additionally, we collected feedback and invited suggestions from the teams of the specialised outpatient units for ASD and IDD and the Klinik am Greinberg. Interventions that directly target children’s CB include, for example, structuring the environment according to the principles of TEACCH (Häußler, 2005; Virues-Ortega et al., 2013), contingency management, and providing effective prompts based on Stepping Stone’s Triple P (Naumann et al., 2007) and the Therapy Program for Children with Hyperkinetic and Oppositional Problem Behaviors [THOP; (Döpfner et al., 2013; Kinnen et al., 2016)], emotion regulation according to the principles of dialectical behaviour therapy [DBT; (Elstner, 2012; McNair et al., 2017)], and the creation of favourable living conditions based on Grawe’s model of basic psychological needs (Grawe, 2000). Interventions strengthening caregivers’ resources are based on the Parenting Stress Model by Abidin (1992), e.g., emotion regulation according to DBT (Fleischhaker et al., 2010; Reicherzer and Bohus, 2017; von Auer and Bohus, 2017), stress management (Kaluza, 2011; Stächele et al., 2020), self-care and resource activation (Lemper-Pychlau and Schneider-Blümchen, 2013; Koppenhöfer, 2018; Reddeman, 2020), mindfulness and relaxation (Li et al., 2018; Weitlauf et al., 2020) and the satisfaction of basic physical needs (Millstein et al., 2020).

Psychoeducational texts were written by HB and JG with support from RM. Subsequently, all texts were converted into plain language by three students of our cooperation partner Prof. Ratz from the Chair of Special Education IV – Education for People with Developmental and Intellectual Disabilities. Those plain language texts were then proof-read by JG, HB and RM to ensure the conservation of meaning.

#### Behaviour analysis process with ProVIA-Kids

ProVIA-Kids (Figures 2, 3) systematically explores the aforementioned potential risk factors for CB via the behaviour analysis algorithm, on the basis of which caregivers are provided with appropriate recommendations.

After challenging behaviour has occurred, caregivers use the app to conduct a behaviour analysis (5–10 min). The algorithm inquires after the child’s mood, aversive tension, frustration, pain/illness, changes in the child’s schedule, nature of the situation (predictability/novelty, degree of structuredness, transition situations, sensory conditions, presence of certain individuals/group situations), prior demands made to the child and reinforcement conditions (positive and negative consequences of the CB). The algorithm assesses the presence of each factor via a single- or multiple-choice question. Each possible answer is defined as either “pathological” (indicating the factor was present in the situation) or “uncritical” (factor not present in the situation). For example, the algorithm asks “Did the situation involve a transition?” If the caregiver answers “Yes” (= pathological answer), cursive for *transition situation* is saved as a situation-specific contributing factor. Subsequently, the user gets a summary of all identified potential risk factors for the behaviour in a given situation. For each identified factor, users get a) an explanation of why this factor is challenging for people with ASD and/or IDD and why it can lead to CB and b) brief recommendations to mitigate that factor in the future (reading time: 5 min). For each brief recommendation, a corresponding extensive psychoeducational chapter is available, in which more detailed information can be found (reading time: 15–30 min).

If the user affirms the presence of, e.g., 5 risk factors in a given situation, they will be presented with a summary list of those 5 factors. If they click on a factor, e.g., sensory hypersensitivity/hyposensitivity, they can read a short explanation and recommendation for this factor. These short recommendation texts contain links to the corresponding extensive chapter(s) with more in detail explanations and strategies. Each affirmative response (“Yes, the risk factor was present”) triggers the corresponding recommendation. The current algorithm doesn’t depend on response patterns.

All user-conducted behaviour analyses are stored in the app to allow for subsequent in-depth study and a review of changes over time. Across all completed analyses, the frequencies of each risk factor are displayed graphically, so user can identify the most common – and likely most important – ones.

Caregivers can also create a profile for each child and for themselves (5–10 min each). The profile items assess cross-situational risk factors as possible triggers or aggravating factors for CB. The child’s profile addresses e.g., unmet physical needs, lack of communication skills or a general lack of structure. The caregiver profile comprises factors influencing the child’s behaviour (e.g., negative attitudes toward the child, dysfunctional attribution of the child’s behaviour) and an assessment of the caregiver’s resources (e.g., depressive symptoms, level of support). The app also provides feedback on all cross-situational factors for which the caregiver indicated a problem and provides corresponding recommendations.

Users can study psychoeducational chapters independently of the behaviour analyses via the menu and mark them as favourites.

The app also includes the option to enter daily assessments of the caregiver’s mood, the amount of self-care and app use and the level of experienced stress due to the CB (5 min). Users can view those four parameters in a graph displaying the previous 7 or 30 days. This allows users to visually recognise relationships, e.g., between increased self-care and improvements in their mood, or between the degree of their app use and a reduction in stress due to the CB. Supplementary Tables 1 and 2 outline the intervention components in greater detail.

### Technical specifications for the ProVIA-Kids app

The ProVIA-Kids app is a cross-platform application developed based on the Ionic framework and, thus, can run on both mobile operating systems, *Apple iOS* and *Android*. The user interface is based on web components developed using the *Vue3* framework. The app provides an extensive web component library (e.g., for buttons, input fields, navigation bars, text containers, or plots) for the user interface, whose implementations can be reused.

**FIGURE 4:**
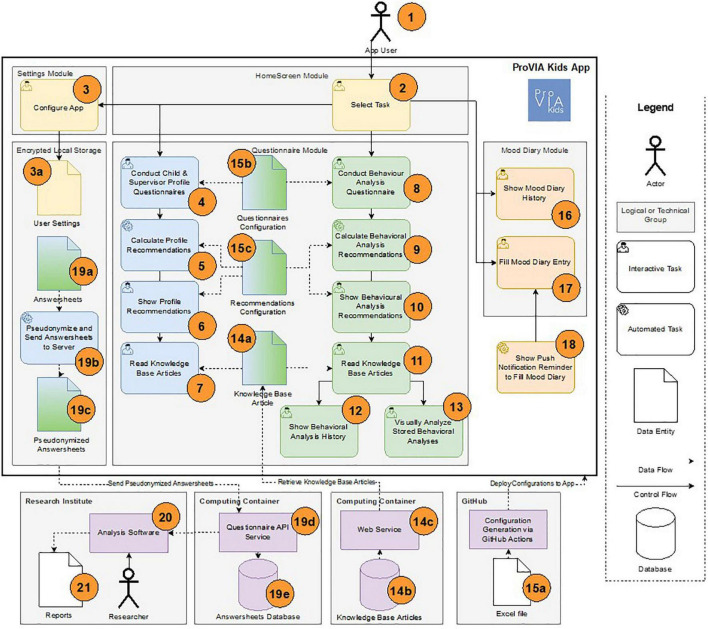
Schematic architecture and workflow of the ProVIA-Kids app.

The app consists of elements grouped into five logical modules (see Figure 4): *HomeScreen*, *Settings*, *Encrypted Local Storage*, *Questionnaire Module*, and *Mood Diary*. The app user (see Figure 4-1) can select various functions on the home screen (see Figure 4-2), including the possibility to configure the app, for example, to delete user data or to change the language (see Figure 4-3). These settings are saved to an encrypted local data storage (see Figure 4-3a). Data storage is implemented using a data access layer that transparently encrypts all data using *Advanced Encryption Standard* (AES) and stores it in an SQLite database. Therefore, the AES symmetric key and initialization vector are generated upon first app start and stored in a secure keychain the mobile operating system provides.

The app’s primary function is to fill out questionnaires, evaluate them automatically, and provide further assistance to users. These functions are grouped together in the Questionnaire Module (see Figure 4–4, 13, 14a, 15b, 15c). For example, users can fill out a profile questionnaire for themselves or the children in their care (see Figure 4-4). Configurable rules (see Figure 4–15c) calculate value spaces from the given answers, which in turn can be assigned to different recommendation texts (see Figure 4–5) and displayed to the user (see Figure 4–6). For example, for a question about the number of hours of sleep, the answer given can be assigned to one of the two recommendation texts “insufficient sleep” or “correct amount of sleep” (also depending on age, for example). The user can also access further information independently of the displayed recommendations via the knowledge section containing articles on different topics (see Figure 4–7). In addition to profile questionnaires, the app also offers a questionnaire for behavioural analysis of a situation with a child in care (see Figure 4–8). For this questionnaire, recommendations can also be calculated and displayed to the user (see Figure 4–9, 10), and further articles can be offered (see Figure 4–11). Already completed questionnaires and their recommendations can be recalled in a list-based history view (see Figure 4–12). The app also offers visual feedback on all behavioural analyses grouped according to the answers given (see Figure 4–13). For example, it can be quickly identified if a particular behaviour only occurs at a specific time of day, during certain activities, or only with specific users.

Knowledge Base articles (see Figure 4–14a) offered in the app (see Figure 4–7, 11) are stored in an external database formatted as Markdown documents (see Figure 4–14b) and can be delivered to the app via a web interface (see Figure 4-14c). Therefore, a developed Markdown parser maps the articles and their contents (e.g., text or images) during display (see Figure 4–7, 4–11).

App questionnaires are defined in Excel files and can thus be quickly customised by non-technical personnel (see Figure 4-15a). These Excel files are stored in a GitHub repository, are converted to JSON configuration files by a GitHub Action when modified, and are added to a new build of the app (see Figure 4–15b). Recommendations are generated based on JSON configuration files (see Figure 4–15c). These include a mapping from answer value spaces (“conditions”) to Markdown documents. For example, a recommendation for a child’s mood in behaviour analysis is displayed if the following condition is evaluated to “true” by a developed expression parser: answer[“child-mood”]===“good” && answer[“child-mood-good”]!==“relaxed.”

The app also lets the user enter their daily mood in a mood diary (see Figure 4-17) and display it in a 7- or 30-days view (see Figure 4-16). In addition, the app can display a daily reminder to fill out the mood diary as an app push notification with the user’s consent (see Figure 4-18).

Questionnaires completed by the user (i.e., profile questionnaires, behavioural analysis questionnaires, and mood diary questionnaires) are stored locally as answersheets in encrypted storage (see Figure 4-19a). Completed questionnaires are pseudonymized (see Figure 4–19b, 19c), then sent transport-encrypted to a REST API service (see Figure 4-19d) (Pryss et al., 2018; Vogel et al., 2021) and stored in a relational database (see Figure 4–19e). The API service and the database are hosted in the UKW data center. Researchers of the participating research institutes can evaluate the stored questionnaires with the help of analysis software (see Figure 4–20) and generate reports (see Figure 4–21).

The app follows a model-driven approach: the individual pages or modules are implemented as web components in TypeScript and Sass or CSS, while the control flow logic, questionnaires, recommendations, and associated knowledge base articles can be variably adapted to the needs of the study to be conducted using various JSON-based configuration files. Hence, questionnaires and recommendations are dynamically generated and displayed based on users’ answers.

### Primary and secondary outcomes

#### Primary outcome

The “Eltern-Belastungs-Inventar” [EBI (Tröster, 2011), the German version of the Parenting Stress Index] measures various facets of parental stress with 48 items. In addition to the EBI total scale, the questionnaire contains five subscales that capture distress sources located in the child (distractibility/hyperactivity of the child, acceptability, demandingness, adaptability, and mood), and seven subscales to capture impairment in parental functioning (attachment, isolation, competence, depression, health, role restriction, spouse/parenting partner relationship). The questionnaire shows an excellent internal consistency for the total scale (Cronbach’s Alpha, α = 0.95), the child subscale (α = 0.91) and the parent subscale (α = 0.93. The EBI shows good test-retest-reliability for the total scale (*r* = 0.87) and the subscales (child subscale: *r* = 0.85 and parent subscale: *r* = 0.87) (Irlbauer-Müller et al., 2017).

#### Secondary outcomes (exploratory)

● The intensity of the child’s challenging behaviour (aggression toward others, aggression toward objects, auto-aggression, screaming, refusal, cursing) is assessed on a 5-point scale (0 = not applicable, 5 = very pronounced). The frequency of CB in those four categories is also recorded.
● Caregiver mood, stress level, self-care and app use are recorded daily via an ecological momentary assessment on a 5-point scale in the app. Means for each week are calculated.
● The “Fragebogen zum Kompetenzgefühl von Eltern” (FKE (Miller, 2001), German Version of the Parenting Sense of Competence Scale) uses 16 items to measure two aspects of parents’ self-esteem: satisfaction with the parenting role (frustration, feelings of failure, motivation) and sense of self-efficacy (competence, problem-solving skills, success). The internal consistency for the English version is acceptable (α = 0.76) and the test-retest-correlations range between *r* = 0.46–0.82 (Gibaud-Wallston and Wandersman, 1978; Johnston and Mash, 1989).
● The short form of the “Erziehungsfragebogen” (EFB-K (Naumann et al., 2010), German version of the Parenting Scale) captures the parental reaction to challenging behaviour with 13 items. Of particular interest is contingency management, which is supposed to be influenced by the app. The internal consistency is acceptable (α = 0.76) (Miller, 2001).

#### Additional parent variables for sample description

● The short form of the “Resilienz-Fragebogen” [RS13 (Leppert et al., 2008)] measures caregivers’ coping ability. The two factors Personal Competence and Acceptance of Self and Life are captured by 13 items. The internal consistency is excellent (α = 0.90) and the test-retest-reliability is good (*r* = 0.61)
● The German version of the “Emotion Regulation Questionnaire” [ERQ (Abler and Kessler, 2009)] measures self-reported preference for the emotion regulation strategies *reappraisal* and *suppression*. The questionnaire contains ten items on dealing with positive and negative emotions. The internal consistency ranges between α = 0.68 and 0.82.
● The “Fragebogen zur Erfassung von Ressourcen und Selbstmanagementfähigkeiten” [FERUS (Jack, 2007)] comprises seven scales (motivation for change, self-observation, active and passive coping, self-efficacy, self-verbalisation, hope, social support). It measures health-related resources and self-management skills with 66 items. The questionnaire has a good to excellent internal consistency (α = 0.86–0.93) and a good test-retest-reliability (*r* = 0.66–0.86) (Znoj and Baumgartner, 2008).
● For the assessment of psychiatric symptoms in caregivers, the Symptom Checklist-90-R [SCL-90-R (Derogatis, 1977)] was used. The questionnaire contains 90 items and describes in 9 scales the domains somatization, obsessive-compulsive disorder, interpersonal sensitivity, depression, anxiety, hostility, phobic anxiety, paranoid ideation, and psychoticism. The questionnaire has excellent internal consistency (α = 0.96–0.98) and a very good test-retest-reliability (*r* = 0.79–0.90, for the interval of 1 week) (Franke, 2002).

#### Additional child variables for sample description

● The “Verhaltensfragebogen bei Entwicklungsstörungen” (VFE (Steinhausen and Winkler Metzke, 2005), German version of the “Developmental Behaviour Checklist” [Einfeld and Tonge, 1995)] measures a wide range of challenging behaviour and emotions in children with developmental disabilities with 96 items. The questionnaire contains five scales (disruptive/antisocial, self-absorbed, communication disturbance, anxiety, social-relating). The questionnaire shows good internal consistency for four scales (α = 0.69–0.90) with the exception of the anxiety subscale (α = 0.58). The test-retest-reliability is high (between *r* = 0.83 and *r* = 0.89, for the interval of 1.8 years).
● The Sensory Profile 2 [SP 2, (Dunn, 2017)] measures sensory processing with 38 items. The questionnaire shows good internal consistency (between α = 0.72 and α = 0.91).
● The “Fragebogen zur Sozialen Kommunikation” [FSK, (Bölte and Poustka, 2005)] assesses abnormal social interaction and communication patterns and stereotypical behaviour with 40 items. The internal consistency of the total scale (α = 0.83) and the test-retest-reliability (*r* = 0.76, for intervals between 6 months and 2 years) are good.

#### Control variables

● As control variables, participants’ treatment motivation, treatment expectancy, and readiness to change are assessed using the “Fragebogen zur Erfassung der Veraenderungsbereitschaft” (FEVER (Hasler et al., 2003), German Version of the University of Rhode Island Change Assessment Scale). FEVER captures the temporal-motivational dimension of the change process with 24 items. The questionnaire shows good internal consistency for the scales (between α = 0.72 and α = 0.86).

#### Evaluation of the app

To evaluate the quality of the psychoeducational content, user will be asked to provide feedback for each intervention module on the comprehensibility and scope of the information and frequency of use of the recommendations. The app’s technical aspects are evaluated using the Mobile Application Rating Scale: user version (uMARS). The uMARS shows an excellent internal consistency (α = 0.90) (Stoyanov et al., 2016). The participants are also asked to provide information about points of criticism and suggestions for improvement regarding the content of the app and the app in general in an open format.

For an overview of all measures used throughout the study, please refer to Table 2.

**TABLE 2:** Instruments and measurements.

		Baseline T0	Post intervention T1	Follow-up T2
**Caregiver variables**				
Sociodemographic questionnaire	Sociodemographic data			
EBI	Caregiver stress	X	X	X
RS13	Resilience	X		
ERQ	Emotion regulation	X		
FERUS	Ressources and self-management	X		
FKE	Parental Competence	X	X	X
EFB-K	Parenting scale	X	X	X
SCL-90-R	Psychiatric symptoms	X		
Mood	Daily scores (assessed via app)	during intervention		
Caregiver burden	Daily scores (assessed via app)	during intervention		
**Child variables**				
Base data	Prior treatments, IQ, CGI			
Challenging behaviour	Frequency/severity of challenging behaviour	X	X	X
VFE	Challenging behaviour	X		
SP2	Sensory Processing	X		
FSK	Social communication (ASS)	X		
ADOS	ASS symptoms (observation)			
ADI	ASS symptoms (parent interview)			
**Evaluation of treatment**				
FEVER	Readiness for change	X		
Treatment expectation		X		
Treatment motivation		X		
Adverse events			X	X
Treatment satisfaction			X	
uMARS			X	
**User data**				
Evaluation of intervention modules				

#### Sample size and power calculations

The calculation of the sample size (software: G*Power Version 3.1) is based on the primary outcome “Change in total score of EBI from T0 (baseline) to T1 (after treatment; 8 weeks)” using a two-sided *t*-test with a power of 80% at a significance level of 5%. The expected effect size is based on the following considerations: A parent training programme for parents of children with intellectual developmental disorder and challenging behaviour showed an effect size of *d* = 0.63 (η = 0.09) for the reduction of parental stress experience (Hudson et al., 2003). Apps as stand-alone procedures for the treatment of depressive symptoms (comparable to resource building components in ProVIA) meta-analytically show an effect size of *d* = 0.33 (Weisel et al., 2019). Based on the assumption that app-based interventions generally produce effects of lower strength, but that ProVIA contains resource-building interventions for caregivers in addition to parent training components, we assume an effect size of *d* = 0.6. We will need *N* = 19 participants to detect an effect of this size and aim at a sample size of *N* = 25 to compensate for dropouts.

### Statistical analyses

All statistical analyses will be performed with IBM SPSS Statistics Version 26. The primary analysis is based on an intention-to-treat approach, i.e., participants will be analysed, irrespective of whether they discontinued the treatment or other protocol violations are revealed. Participants are included if treatment was started. Changes in the primary outcome (EBI score from T0 to T1 and T0 to T2) will be evaluated with paired-samples *t*-tests. For additional exploratory analyses, possibly relevant covariates are assessed in a repeated measures analysis of covariance. Changes in secondary outcomes are examined using paired *t*-tests. The relationship between parental resilience (RS13), emotion regulation (ERQ) and resources and self-management skills (FERUS) and the degree of change in parental stress experience (difference in EBI total scale from T0 to T1) is tested using correlation analyses in an exploratory manner. Intention-to-treat refers solely to the treatment, i.e., the use of the ProVIA-Kids app. We will analyse data from all participants from which we have T0 and T1 or T0 and T2, respectively. Study drop-outs with whole missing assessments will be excluded from analysis. Missings in individual questionnaires will be handled according to the respective manual. We will tolerate max. 10% missing items. Ten percentage of missing items in total and 1 missing item per scale. If there are no specific instructions, we will use the mean of the respective scale to replace an individual missing value. We expect a very low degree of missingness and drop-outs. However, we will compare complete drop-outs (no T1 or T2 assessment) to the completer group in terms of initial parental stress, initial child problem behaviour, sociodemographic variables (employment, single parenthood, support), and diagnosis to get an idea about factors leading to drop-outs.

## Discussion

The aim of the ProVIA project is to investigate the efficacy of a digital behaviour analysis tool addressing CB in children with ASD and/or IDD for reducing the caregiver’s stress burden.

Behaviour analyses are the foundation of any cognitive-behavioural intervention. They are used to identify the causes and maintaining conditions of dysfunctional behaviour, the modification of which allows for behavioural change. Behaviour analysis is an effective method for addressing CB in patients with ASD and/or IDD (Dunlap and Fox, 2011; NICE, 2015). In addition to modifying the behaviour, they also positively influence caregivers’ attitudes toward the child and the behaviour, e.g., by attributing CB less to negative traits of the child and more to circumstances and a need for support. However, caregivers are largely dependent on the availability of a therapy slot for the child to be guided in the use of behaviour analysis by the child’s therapist. Considering the scarcity of therapy places and general resources for caregivers and the high levels of stress caregivers experience due to CB (Jungbauer and Meye, 2008; Giovagnoli et al., 2015; Geissler et al., 2021b), ProVIA addresses a highly relevant issue.

To the best of our knowledge, the ProVIA-Kids app is the first attempt to translate a comprehensive behaviour analysis into a digital and automatized format. With the app, caregivers of children with ASD and/or IDD have at their disposal a free of charge, low-threshold, practice-oriented tool that can support them in their everyday life dealing with CB. The app promotes an understanding of the causes of the behaviour and provides strategies for handling and preventing CB.

For the study, the app will be tested by parents as caregivers. Detailed feedback on both the content and the technical aspects will be collected from study participants. If results from the study indicate that digital automated behaviour analysis is feasible and effective, this tool can support a broad group of caregivers, e.g., therapists, teachers or staff in kindergartens and residential facilities.

### Challenges concerning automated behaviour analysis

Despite the opportunities this app offers, however, there are also potential risks we need to consider concerning automated behaviour analysis. It is unclear whether automated behaviour analysis can adequately identify and map the individual causes of CB considering the complexity of human decision-making processes. While therapists can freely explore possible causes of a given behaviour in conversations with caregivers and through observation of the patient, the app’s algorithm required a pre-selection of risk factors, which are then queried for each situation. We based the selection of risk factors on the guidelines for ASD and IDD as well as clinical experience from our specialised inpatient and outpatient treatment settings. Nevertheless, behaviour analysis is a method that requires a high degree of individualisation. In contrast to the app, therapists can, for example, explore circumstances in a more targeted way, specifically observe certain aspects and spot inconsistencies. It is still unclear whether breaking CB down to a few common risk factors can result in meaningful interventions for the individual patient.

Another fundamental question is whether lay people can successfully conduct a behaviour analysis without the support of clinical experts: Can they adequately answer the questions comprising the behaviour analysis? Can they implement the rather complex interventions? Or are the demands too high? In order to support users in understanding the mode of action/logic of a behaviour analysis and in using the app, we designed an app user guide, which is presented prominently on the home screen. However, it is possible that a therapist-guided introduction to the method and the joint exemplary performance of some analyses are requirements for sensitising caregivers to the relevant information and facilitating the meaningful interpretation of the behaviour analysis results.

### Potential harms for patients related to the self-help approach

Mental health interventions carried out by lay people in a self-help format without professional supervision (e.g., by therapists) place a lot of responsibility on the users. The absence of external feedback confers certain risks for patients. If interventions are used incorrectly or for the wrong purposes, there is no external control instance. To prevent harm from incorrect app use, we have taken several risk mitigation measures. We clearly defined the target group (children with ASD or IDD aged 3–11 years) and the forms of CB that can be addressed by the app (predominantly aggressive behaviour). The specific age range was chosen, because comorbidities and the manifestation of CB vary with age. For example, the onset of puberty often represents a marked change and therefore the use of the app as a stand-alone method is not appropriate. To prevent comprehension problems all texts are written in plain language. Additionally, throughout the psychoeducational chapters and recommendations caregivers are encouraged to involve specialists from different professions for issues exceeding the scope of the app. This was done to minimise risks associated with user frustration due to user errors or excessive demands. Furthermore, we wanted to avoid suggesting that even the most serious behavioural issues can be solved via an app alone. Finally, users are informed about general risks of behaviour modification that also occur in classical in-person behaviour therapy, such as an initial increase in CB or interpersonal conflicts.

### General challenges related to mental health applications

Beyond the technical and content-related aspects, there are some fundamental challenges with regard to the medium of MHAs, which will be briefly discussed here. In their recent review, Bauer and colleagues discuss various risks associated with MHA (Bauer et al., 2020). There seems to be a group of people who do not use health applications (HA) for various reasons or do not find them attractive (Krebs and Duncan, 2015) due to e.g., a general lack of interest, (hidden) costs or data protection concerns. ProVIA-Kids addresses the data protection risks through pseudonymisation and the storage of data on a secure server at the University Hospital of Würzburg. The project’s public funding and the resulting independence from private-sector interests or advertising partners contributes to a high standard of data protection and ensures that the app remains completely free of charge. However, even among people who are open to using HAs, the use of the HAs or interventions to be carried out independently (including online programmes) in general shows highly variable and often low retention rates, especially outside the study context. Since motivation and commitment are essential, ProVIA-Kids was designed to be attractive for users, e.g., by addressing them personally and using interactive elements such as a mood diary.

### Outlook

There is a high demand for low-threshold interventions supporting caregivers of children with ASD/IDD who display CB. Irrespective of the results regarding the efficacy of the ProVIA-Kids app for reducing parental stress and CB in children, the study will provide interesting insights into the needs of the target group in terms of digital interventions. If results are promising with regard to the efficacy of the ProVIA-Kids app, the principle of algorithm-based behaviour analysis can be transferred to other mental disorders and thus represents a valuable tool in the stepped-care treatment of mental illnesses.

## Dissemination

Results will be communicated to the public via publication in peer-reviewed journals, presentation at scientific conferences, and other meetings with an audience with an interest in psychotherapy research, press releases, and self-help organizations. The study protocol will be made available upon request.

## Data availability statement

Original data will be made available on request after the publication of the main results. Further inquiries can be directed to the corresponding author.

## Ethics statement

The study involving human participants was reviewed and approved by the ethics committee of the Medical Faculty of the University of Würzburg, Germany (AZ 233/21-me). If changes to the protocol are made, ethics approval for the new version will be obtained and the registration will be updated. Written informed consent to participate in this study was provided by the participants’ legal guardian/next of kin.

In order to participate in the study, all persons holding custody of the child are first briefed extensively either in person or via telephone about the study procedures and goals. The family then receives the information in written form prior to the first appointment. At the beginning of the screening appointment, a member of the study coordination team is present to answer any open questions and assess whether all parties voluntarily participate. Then written informed consent is obtained and the screening begins. Participants can withdraw their consent at any time without giving reasons and without incurring any disadvantages as a result. Furthermore, the study can be discontinued by the study coordinators if an adverse event occurs or if a different type of treatment is necessary for the well-being of the study participant. The study coordinators will inform the study participants in a personal conversation as soon as the circumstance leading to the discontinuation becomes known. In these cases, support measures (e.g., follow-up treatment) will be initiated.

## Author contributions

JG was the coordinator of the ProVIA study, wrote the proposal to acquire funding, was involved in designing the trial and the interventions, and wrote the first draft of the manuscript. HB was critically involved in the planning and the conduct of the study, designing the trial and interventions, and in writing the first draft of the manuscript. RM was critically involved in planning and conduct of the study and in finalising the manuscript. KK and MG were responsible for programming the ProVIA-Kids application and critically revised the manuscript. JS was responsible for developing the backend application for the ProVIA-Kids application and critically revised the manuscript. CR was critically involved in the planning and the conduct of the study, designing the trial and interventions, and in finalising the manuscript. RP was involved in designing the smartphone application and critically revised the manuscript. MR was involved in planning, funding acquisition, and critically revised the manuscript. All authors contributed to the article and approved the submitted version.
