# Supplementary material for: Smartphone-based behaviour analysis for challenging behaviour in intellectual and developmental disabilities and autism spectrum disorder – Study protocol for the ProVIA trial
Source: Front Neurosci. 2022 Oct 13;16:984618. doi: 10.3389/fnins.2022.984618 (PMC9610118; doi:10.3389/fnins.2022.984618)
Supplement: Supplementary file 1 [file Data_Sheet_1.pdf]

Studienteilnehmer-Information und Einwilligungserklärung zur Teilnahme an der Studie

„Problemverhalten verstehen und vorbeugen bei Intellektueller Entwicklungsstörung und Autismusspektrumstörungen (ProVIA)“

Studienteilnehmer-Information für Sorgeberechtigte

Liebe Sorgeberechtigte,

wir möchten Sie fragen, ob Sie bereit sind, mit Ihrem Kind an der nachfolgend beschriebenen Studie teilzunehmen. Die Aufklärung über die Studie erfolgt in einem ausführlichen Gespräch. Ihre Teilnahme an der Studie ist vollkommen freiwillig. Bevor Sie sich damit einverstanden erklären, soll die vorliegende Studienteilnehmer-Information Ihnen erläutern, warum wir die Studie durchführen. Bitte lesen Sie den folgenden Text als Ergänzung zum Informationsgespräch mit einem Studienmitarbeiter oder einer Studienmitarbeiterin sorgfältig durch und zögern Sie nicht, Fragen zu stellen.

1. Warum wird diese Studie durchgeführt?

Kinder mit Intelligenzminderung und/oder Autismus-Spektrum-Störungen zeigen sehr häufig herausfordernde Verhaltensweisen, die zu einer starken elterlichen Belastung führen können. Gleichzeitig gibt es eine Versorgungslücke im Gesundheitssystem: Viele Familien haben keine Anlaufstelle, da es nur wenige spezialisierte Ambulanzen, Kliniken und Wohneinrichtungen gibt. Sowohl Eltern als auch Therapeut*innen und Mitarbeiter*innen in Heimeinrichtungen fühlen sich oft überfordert.

Das Ziel des Projektes besteht darin, ein digitales Tool (eine Smartphone-App) zu entwickeln, das Betreuungspersonen von Kindern und Jugendlichen mit Intelligenzminderung hilft, die Ursachen von Problemverhalten zu verstehen. Die App gibt Ihnen dann konkrete verhaltenstherapeutisch und pädagogisch basierte Handlungsempfehlungen, wie Sie mit dem Problemverhalten umgehen und es in Zukunft verhindern können.

2. Wie ist der Ablauf der Studie und was muss bei Teilnahme beachtet werden?

Vor Aufnahme in die Studie werden Sie über Wesen, Bedeutung und Zweck der Studie aufklärt. Auch wird über die Rechte (z.B. die Möglichkeit, jederzeit die weitere Teilnahme an dieser Studie abzulehnen) bei einer Teilnahme aufgeklärt. Den Sorgeberechtigten und den Kindern wird ausreichend Zeit für ihre Entscheidung zur freiwilligen Teilnahme eingeräumt.

Nach Ihrer Einwilligung in die Studienteilnahme (mündlich und schriftlich) erfolgt der Studieneinschluss und die Untersuchungstermine werden vereinbart.

Bei der Eingangsuntersuchung (T0) erhalten Sie Fragebögen. Die Fragebögen beziehen sich auf Sie als Betreuungsperson, und andererseits auf das Verhalten des Kindes. Zu Ihnen als Betreuungsperson werden soziodemografische Daten und Angaben zu Ihrem Wohlbefinden, Belastungserleben, Erziehungsverhalten und der Behandlungsmotivation erfragt.

Zusätzlich wird ein kurzes Interview durchgeführt, in dem bisherige Behandlungen und Vorbefunde zur Begabung und gegebenenfalls zur autistischen Symptomatik des Kindes erfasst werden.

Sie erhalten im Anschluss einen Zugang zu der Smartphone-App. Für die Studie sollen Sie die über einen Zeitraum von <u>8 Wochen</u> benutzen.

Bei der ersten Nutzung der App erhalten Sie einen Teilnehmer*innen-Code. Ein Studienmitarbeiter oder eine Studienmitarbeiterin wird sich bei Ihnen melden und diesen Code erfragen. Dadurch können die Daten, welche die App pseudonymisiert (ohne Angabe Ihres Namens oder des Namens Ihres Kindes, d.h. es können keine Rückschlüsse auf Sie gezogen werden) an die Studienmitarbeiter*innen übermittelt, den Daten aus den Fragebögen zugeordnet werden, ohne dass sie mit Ihrem Namen verbunden sind.

Nachdem Sie die App 8 Wochen lang verwendet haben, findet erneut ein Messzeitpunkt statt (T1). Sie erhalten hier Fragebögen, um Veränderungen im Verhalten des Kindes und in Ihrem Wohlbefinden durch die Nutzung der App zu erfassen. Sie werden in einem kurzen Interview zu aktuellen Behandlungen und möglichen Nebenwirkungen der App-Nutzung befragt.

3 Monate nach diesem Termin findet der letzte Messzeitpunkt (T2) statt. Hier wird zum letzten Mal mit Fragebögen erfasst, ob die Veränderungen über einen längeren Zeitraum hinweg stabil sind. Sie werden erneut in einem kurzen Interview zu aktuellen Behandlungen und möglichen Nebenwirkungen der App-Nutzung befragt.

Die ProVIA-App

Die von der Universität Würzburg in unserem Auftrag programmierte App soll Betreuungspersonen von Kindern und Jugendlichen mit Autismus oder kognitiver Entwicklungsstörung dabei helfen, die Ursachen von herausforderndem Verhalten zu erkennen.

Herausforderndes Verhalten ist immer ein Lösungsversuch für ein bestimmtes Problem in einer bestimmten Situation. Verhalten ist veränderbar. Wir wollen Sie dabei unterstützen, die Auslöser des Verhaltens zu erkennen. Wenn Sie die Auslöser kennen, dann können Sie dem Kind helfen, eine andere Lösung für sein Problem zu finden. Damit lässt sich herausforderndes Verhalten reduzieren.

Die App führt mit Ihnen sogenannte Verhaltens-Analysen durch. Eine Verhaltens-Analyse unterstützt Sie dabei, alle möglichen Ursachen des herausforderndem Verhalten zu finden. Das Ziel ist es, schwierige Situationen im Vorfeld zu erkennen und herausforderndes Verhalten zu verhindern.

Im Anschluss an eine Situation, in der es zu Problemverhalten gekommen ist, stellt Ihnen die App Fragen zu möglichen Gründen für das Verhalten. Wenn Sie alle Fragen beantwortet haben, dann

bekommen Sie eine Übersicht der wahrscheinlichen Ursachen, die in der Situation eine Rolle gespielt haben.

Zu jeder dieser wahrscheinlichen Ursache erhalten Sie eine Erklärung: Warum kann das bei Menschen mit Autismus und/oder Intelligenzminderung zu Problemverhalten führen? Außerdem erhalten Sie Handlungsempfehlungen, wie Sie mit der Ursache in Zukunft besser umgehen und dadurch Problemverhalten verhindern können. Alle alten Verhaltens-Analysen sind in der App gespeichert. Sie haben hier die Möglichkeit, sich die Analysen jederzeit erneut anzuschauen und Veränderungen über die Zeit zu beobachten.

Die App enthält ausführliche Wissenskapitel mit Informationen zu 1) Autismus und kognitiven Entwicklungsstörungen, 2) Ursachen und Empfehlungen für Problemverhalten und 3) Ressourcen für die eigene Gesundheit.

1) Um das kindliche Verhaltens besser zu verstehen, erhalten Sie in den Wissenskapiteln zu Autismus und kognitiven Entwicklungsstörungen umfassende Informationen zu Entstehung und Besonderheiten der Störungsbilder. Außerdem wird der Stellenwert günstiger Lebensbedingungen nicht nur für das Wohl des Kindes, sondern auch als Schutzfaktor gegen herausforderndes Verhalten erklärt. Sie erhalten hier Informationen, wie Sie günstige Bedingungen (zum Beispiel: Selbstbestimmung, Kompetenzerleben, Bedürfnisbefriedigung, und gute zwischenmenschliche Beziehungen) für das Kind sicherstellen können. Es werden außerdem mögliche Therapie- und Förderangebote vorgestellt.

2) Ursachen, die bei den Kindern häufig zu Problemverhalten führen, sind zum Beispiel eine mangelnde Strukturierung der Umwelt, Schwierigkeiten im Umgang mit Gefühlen, sensorische Besonderheiten, Überforderung oder unbefriedigte körperliche Grundbedürfnisse. In den Wissenskapiteln zu den Ursachenfaktoren können Sie zum Beispiel lernen, wie Sie für das Kind eine gute Strukturierung von Raum, Zeit und Aktivitäten schaffen. Sie werden angeleitet dem Kind bei der Regulation unangenehmer Gefühle zu helfen, Anspannung zu reduzieren und das Kind dabei zu unterstützen, stressauslösende Situationen zu bewältigen. Sie können Strategien zur Förderung der Beziehung zum Kind anwenden. Außerdem werden Sie angeleitet, die Folgen des kindlichen Verhaltes so zu verändern, dass unerwünschtes Verhalten seltener und erwünschtes Verhalten häufiger wird.

3) Wichtige Ressourcen für Betreuungspersonen sind zum Beispiel Selbstfürsorge, ein guter Umgang mit Stress und schwierigen Gefühlen und Unterstützungsmöglichkeiten bei der Betreuung des Kindes und im Umgang mit schwierigem Verhalten. Sie könne in diesen Wissenskapiteln zum Beispiel lernen, wie Sie Überlastung vermeiden und Widerstandsfähigkeit gegen Stress aufbauen können, indem Sie auf Ihre Grundbedürfnisse achten, Selbstfürsorge betreiben und angenehme Aktivitäten und Entspannung in den Alltag integrieren. Es werden außerdem Achtsamkeits- und Entspannungsverfahren und Strategien zur Steigerung des Selbstwerts und Vorbeugung von Stress vermittelt. Die App Ihnen die Möglichkeit ein Tagebuch zu Ihrer Stimmung und zum Belastungserleben zu führen und angenehme Selbstfürsorge-Aktivitäten in der Kalenderoption zu planen.

Die Inhalte der App sind in einfacher Sprache erstellt, damit die Verständlichkeit für alle Nutzer und Nutzerinnen sichergestellt ist.

3. Wer darf an der Studie teilnehmen?

Betreuungspersonen mit einem Kinde im Alter von 3 bis 11 Jahren, bei dem die Diagnose einer Intelligenzminderung (IQ<70) oder einer Autismusspektrumstörung nach ICD-10 oder DSM-5 vorliegt.

4. Wer ist der Auftraggeber der Studie und wie viele Patienten werden an der Studie teilnehmen?

Die Studie wird vom Bayerischen Staatsministerium für Familie, Arbeit und Soziales gefördert.

Für die Studie möchten wir insgesamt 25 Familien gewinnen. So können wir verlässliche Aussagen über die Eignung des digitalen Interventionstools treffen.

5. Wie lange dauert die Teilnahme an der Studie?

Die Teilnahmedauer an der Studie beträgt ca. 24 Wochen.

6. Welche Risiken bzw. welcher Nutzen ist mit der Studienteilnahme verbunden?

Allen Behandlungen, die in dieser Studie zum Einsatz kommen, sind für die Behandlung etabliert. Die Wirksamkeit in Form einer Smartphone-App als digitale Gesundheitsintervention wurde jedoch bisher nicht genau untersucht. Es ist daher noch unklar, welche Kinder und Sorgeberechtigten von der Behandlungsstrategie am meisten profitieren. Unserer Studie möchte herausfinden, für wen diese Art der Behandlung geeignet ist.

Mögliche Nebenwirkungen sind zum Beispiel Stimmungsveränderungen oder gesteigerte Unruhe. Das Problemverhalten des Kindes kann zunächst zunehmen, wenn Sie Ihr eigenes Verhalten verändern.

Wir können nicht mit Sicherheit sagen, ob Sie und Ihr Kind von der Studienteilnahme profitieren werden. Die gesammelten Informationen aus dieser Studie können jedoch helfen, bei anderen Kindern mit Autismus oder kognitiver Entwicklungsstörung in Zukunft besser zu entscheiden, ob das App-Angebot als unterstützende Maßnahmen zur Anwendung kommen soll.

7. Welche anderen Behandlungsmöglichkeiten gibt es außerhalb der Studie?

Sie müssen nicht an dieser Studie teilnehmen, wenn Sie das nicht wollen. Falls Sie sich gegen eine Studienteilnahme entscheiden, hat das keine Nachteile für die Behandlung Ihres Kindes am Universitätsklinikum Würzburg.

8. Gibt es eine Patientenversicherung?

Da im Rahmen der Studie keine medikamentöse Behandlung vorgesehen ist, die therapeutischen Maßnahmen etabliert sind und deshalb keine studienspezifischen Risiken

erwartet werden, ist keine spezielle Patientenversicherung zur Absicherung eines studienspezifischen Risikos vorgesehen.

9. Entstehen für mich Kosten durch die Teilnahme an der Studie? Erhalte ich eine Aufwandsentschädigung?

Durch die Teilnahme an dieser Studie entstehen für Sie oder Ihr Kind keine zusätzlichen Kosten. Eine Aufwandsentschädigung ist nicht vorgesehen.

10. Freiwilligkeit der Studienteilnahme

Die Teilnahme von Ihnen und Ihrem Kind an dieser Studie ist freiwillig. Es ist allein Ihre Entscheidung und die Entscheidung Ihres Kindes, teilzunehmen oder nicht. Auch wenn Sie sich zur Teilnahme entscheiden und die Einwilligungserklärung unterschreiben, können Sie und Ihr Kind jederzeit und ohne Angabe von Gründen ihre Einwilligungserklärung zur Teilnahme an der Studie widerrufen. Dies hat keinerlei Auswirkung auf die weitere Behandlung Ihres Kindes oder Nachteile für Sie oder Ihr Kind.

Wenn Sie und / oder Ihr Kind die Einwilligung zur Studienteilnahme widerrufen, wird ein*e Studienmitarbeiter*in um die Einwilligung bitten, eine Abschlussuntersuchung durchzuführen und die Daten in einem schriftlichen Bericht zu sammeln. Die Teilnahme an der Abschlussuntersuchung ist freiwillig. Ohne Ihre Zustimmung und die Ihres Kindes werden der Studienakte keine neuen Daten über Ihr Kind hinzugefügt. Auf Ihren Wunsch werden die bisher über Sie und Ihr Kind erhobenen Daten gelöscht bzw. vernichtet.

Selbst wenn Sie Ihre Einwilligung geben, wird die Entscheidung Ihres Kindes, nicht an der Studie teilzunehmen bzw. die Teilnahme an der Studie vorzeitig zu beenden, respektiert.

Ein*e Studienmitarbeiter*in wird Sie über alle neuen Erkenntnisse, die in Bezug auf diese Studie bekannt werden, und für Ihr Kind von wesentlicher Bedeutung sein könnten, umgehend informieren. Auf dieser Basis können Sie dann die Entscheidung zur weiteren Teilnahme an dieser Studie neu überdenken.

11. Kann die Teilnahme an der Studie auch durch Andere vorzeitig beendet werden?

Es ist möglich, dass die Studienverantwortlichen entscheiden, die Teilnahme Ihres Kindes an der Studie vorzeitig zu beenden, ohne vorher Ihre Einwilligung einzuholen. Gründe hierfür können darin bestehen, dass

- Starke Nebenwirkungen auftreten.
- den Erfordernissen der Studie nicht ausreichend nachgekommen wird oder werden kann.
- neue Informationen darauf hinweisen, dass die Behandlung nicht länger sinnvoll ist.

Ein*e Studienmitarbeiter*in wird dann die Situation und die weitere Vorgehensweise mit Ihnen besprechen.

12. Was geschieht mit den Daten?

Während der Studie werden Befunde, die Ergebnisse der Fragebögen und andere personenbezogene Daten von Ihnen erhoben und in der Kinder- und Jugendpsychiatrie des Universitätsklinikums Würzburg (UKW) in der persönlichen Studienakte Ihres Kindes niedergeschrieben oder elektronisch gespeichert. Entsprechend der gesetzlichen Vorgaben erfolgt die Speicherung pseudonymisiert, d.h. ohne Angaben von Namen, Geburtsdatum oder anderen Informationen, durch die Sie oder Ihr Kind direkt identifiziert werden können, sondern nur mit einem Nummern- und / oder Buchstabencode.

Ihr Name, der Ihres Kindes, Ihre Adresse und Telefonnummer werden auf einer zweiten Liste, der sog Zuordnungsliste, vermerkt, die auch den Nummern- und /oder Buchstabencode enthält. Dies ist deshalb notwendig, damit wir die Antworten der Fragebögen und die sonstigen Daten im Verlauf der Studie korrekt zuordnen können. Die Zuordnungsliste wird getrennt von der Studienakte aufbewahrt. Eine Zuordnung der Daten der Studienakte zu Ihrer Person oder zu Ihrem Kind erfolgt nur unter den vom Gesetz vorgeschriebenen Voraussetzungen.

In die Studienakte fließen auch die über die App erhobenen, für die Studie wichtigen Daten (Inhaltsdaten aus Profilen, den durchgeführten Verhaltensanalysen und dem Stimmungstagebuch, Ihr Feedback zu den Wissenskapiteln und Ihre Bewertung der Empfehlungen) ein. Auch diese Informationen werden in der App in pseudonymisierter Form gespeichert und automatisch an einen Server des Instituts für klinische Epidemiologie und Biometrie der Universität Würzburg gesendet. Die Universität Würzburg fungiert dabei als Dienstleister, ist dem UKW gegenüber streng weisungsgebunden und entsprechend vertraglich verpflichtet.

Von dem Server der Universität werden die Daten über eine gesicherte Verbindung in die Studienakte am UKW übermittelt.

In der App werden auch Ihr Benutzername und der von Ihnen optional eingegebene Name des Kindes gespeichert. Diese Daten werden nicht an den Server der Universität und damit auch nicht in die Studienakte übermittelt. Alle Daten in der App werden für immer gelöscht, wenn die App von Ihrem Smartphone gelöscht wird oder Sie in den Einstellungen der App die Inhaltsdaten löschen. Die Daten, die bereits in der Studienakte Ihres Kindes gespeichert sind, sind von der Löschung der Daten in der App nicht betroffen. Protokolldaten zu der App-Nutzung oder Fehlerberichte werden nicht erhoben. Eine detaillierte Aufstellung der verarbeiteten Daten finden Sie in der Datenschutzerklärung der App.

Die Ergebnisse der Studie können in wissenschaftlichen Fachzeitschriften veröffentlicht werden, zudem erhält das Bayerische Staatsministerium für Familie, Arbeit und Soziales als Geldgeber einen Abschlussbericht. Die Veröffentlichungen und Berichte werden keine Daten enthalten, durch die auf die Identität der Studienteilnehmerinnen und -teilnehmer und deren Kinder geschlossen werden kann.

Eine darüberhinausgehende Übermittlung Ihrer personenbezogenen Daten an Dritte findet nur statt, wenn das UKW gesetzlich dazu verpflichtet sind oder Sie vorab in die Datenübermittlung eingewilligt haben. Dies gilt auch für die Datenübermittlung an ein Drittland.

Die während der Studie erhobenen Daten werden nach Studienabschluss, wie gesetzlich vorgeschrieben, im rohen Datenformat für 10 Jahre aufbewahrt. Die Zuordnungsliste mit Ihrem Namen und Ihren Kontaktdaten wird bereits mit Abschluss der Studie datenschutzkonform vernichtet.

Zweck der Datenverarbeitung ist die Durchführung der beschriebenen Studie. Rechtsgrundlage für die Datenverarbeitung im Rahmen der Studie ist die Einwilligung (s.o. Nr. 10, vgl. auch. Art. 6 Abs. 1 Satz 1 Buchst. a sowie Art. 9 Abs. 2 Satz 1 Buchst. a der DSGVO).

Verantwortlich für die Datenverarbeitung in dieser Studie ist:

Universitätsklinikum Würzburg (UKW)
Anstalt des öffentlichen Rechts
Josef-Schneider-Straße 2
97080 Würzburg
Tel.: 0931-201-0
E-Mail: info@ukw.de

Hinweise auf Ihre Betroffenenrechte:

Recht auf Auskunft
Sie haben das Recht, Auskunft über Ihre im Rahmen dieser Studie verarbeiteten personenbezogenen Daten zu verlangen (Art. 15 EU-DSGVO) und eine unentgeltliche Kopie dieser Daten zu erhalten.

Recht auf Datenberichtigung
Sie haben das Recht, die Berichtigung Ihrer personenbezogenen Daten zu verlangen, wenn diese unvollständig oder falsch sein sollten (Art. 16 EU-DSGVO).

Recht auf Sperrung (Einschränkung der Datenverarbeitung)
Sie haben das Recht, eine Einschränkung der Verarbeitung (= Sperrung) Ihrer personenbezogenen Daten zu verlangen (Art. 18 EU-DSGVO), z. B. wenn Sie die Richtigkeit der betreffenden Daten für die Dauer der Überprüfung dieses Anliegens beanstanden.

Recht auf Widerruf (Datenschutzrechtliches Widerrufsrecht)
Sie haben jederzeit die Möglichkeit, ohne Angabe von Gründen die Einwilligung zur Verarbeitung Ihrer personenbezogenen Daten zu widerrufen (Art. 7 Abs. 3 EU-DSGVO). Die Rechtmäßigkeit der bis zum Widerruf erfolgten Verarbeitung Ihrer personenbezogenen Daten wird dadurch nicht berührt.

Recht auf Datenlöschung
Sie haben ein Recht auf Löschung Ihrer personenbezogenen Daten (Art. 17 EU-DSGVO), z.B. wenn die Daten für die verfolgten Zwecke nicht mehr benötigt werden. Ein Recht auf Datenlöschung besteht also nicht, wenn durch dessen Inanspruchnahme die wissenschaftlich korrekte Durchführung des Forschungsvorhabens unmöglich gemacht oder ernsthaft beeinträchtigt würde.

Wenn Sie eines dieser Rechte in Anspruch nehmen möchten, wenden Sie sich am besten an die unter 13. genannten Personen.

Recht auf Beschwerde bei einer Aufsichtsbehörde
Zudem haben Sie das Recht auf Beschwerde bei einer Datenschutzaufsichtsbehörde, wenn Sie der Ansicht sind, dass die Verarbeitung Ihrer Daten datenschutzrechtlich nicht zulässig ist. Für das UKW zuständig ist der Bayerische Landesbeauftragte für den Datenschutz, Postfach 22 12 19, 80502 München.

Bei allen **Fragen zum Datenschutz** können Sie sich zudem an den Datenschutzbeauftragter des Universitätsklinikum Würzburg, Josef-Schneider-Str. 2, 97080 Würzburg, E-Mail: datenschutz@ukw.de, wenden.

13. An wen wende ich mich bei weiteren Fragen?

Für weitere Fragen im Zusammenhang mit dieser Studie stehen Ihnen die Studienmitarbeiter*innen gerne zur Verfügung. Auch Fragen, welche die Rechte Ihres Kindes als Patient und Teilnehmer an dieser Studie oder die Aufbewahrung bzw. Vernichtung von persönlichen Daten betreffen, werden Ihnen gerne beantwortet. Wenn Sie, Ihr Kind oder Ihre Angehörigen Fragen oder Bedenken zu dieser Studie, zu möglichen Risiken und unerwünschten Nebenwirkungen der eingesetzten Behandlungsformen oder zu Patientenrechten haben, wenn Sie zusätzliche Informationen wünschen oder studienbezogene Schädigungen melden möchten, können Sie und Ihr Kind jederzeit ein Mitglied des Studienpersonals kontaktieren.

**Name der Kontaktperson:**	**Erreichbar unter:**
Dr. Julia Geissler<br>Diplom-Psychologin<br>Psychologische Psychotherapeutin<br>(Verhaltenstherapie)	Universitätsklinikum Würzburg<br>Zentrum für Psychische Gesundheit<br>Klinik und Poliklinik für Kinder- und Jugend-psychiatrie, Psychosomatik und Psychotherapie<br><br>Tel.: +49 – 931 – 201 78 710<br>Fax: +49 – 931 – 201 78 040<br>E-Mail: Geissler_J@ukw.de
Hanna Buchholz<br>Sonderpädagogin (Staatsexamen)<br>Kinder- und Jugendlichen-Psychotherapeutin in Ausbildung	Universitätsklinikum Würzburg<br>Zentrum für Psychische Gesundheit<br>Klinik und Poliklinik für Kinder- und Jugend-psychiatrie, Psychosomatik und Psychotherapie<br>E-Mail: Buchholz_H@ukw.de
Rina Meerson<br>B.Sc. Psychologie	Universitätsklinikum Würzburg<br>Zentrum für Psychische Gesundheit<br>Klinik und Poliklinik für Kinder- und Jugend-psychiatrie, Psychosomatik und Psychotherapie<br>E-Mail: Meerson_R@ukw.de

„Problemverhalten verstehen und vorbeugen bei Intellektueller Entwicklungsstörung und Autismusspektrumstörungen (ProVIA)“

Einwilligungserklärung

.... geb. am ....

Name des Kindes in Druckbuchstaben

.... ………………………………………………………

Name des ersten Sorgeberechtigten Name des zweiten Sorgeberechtigten

Ich bin / Wir sind in einem persönlichen Gespräch durch den / die Studienmitarbeiter*in

....

Name des / der Studienmitarbeiter*in

ausführlich und verständlich über die geplante Behandlung sowie über Wesen, Bedeutung, Risiken und Tragweite der Studie aufgeklärt worden. Ich habe darüber hinaus den Text der Studienteilnehmer-Information sowie die hier nachfolgend abgedruckte Datenschutzerklärung gelesen. Ich hatte / Wir hatten die Gelegenheit, mit dem / der Studienmitarbeiter*in über die Durchführung der Studie zu sprechen. Alle meine / unsere Fragen wurden zufrieden stellend beantwortet.

Ich hatte / wir hatten ausreichend Zeit, mich / uns zu entscheiden.

Mir / Uns ist bekannt, dass ich / wir jederzeit und ohne Angabe von Gründen meine / unsere Einwilligung zur Teilnahme an der Studie widerrufen kann / können (mündlich oder schriftlich), ohne dass daraus Nachteile für die therapeutische Behandlung entstehen.

Wir willigen ein, mit unserem Kind an der oben genannten Studie teilzunehmen.

Ebenso willigen wir auch in die Datenverarbeitung, wie sie in der Information dargestellt ist, ein.

- ☐ Ein Exemplar der Studienteilnehmer-Information und der Einwilligungserklärung haben wir erhalten. Ein Exemplar verbleibt im Prüfzentrum.

- ☐ Wir entbinden das Studienpersonal von der Schweigepflicht gegenüber dem / der behandelnden Kinder- und Jugendpsychiater*in bzw. der Einrichtung, in welcher die Routinebehandlung stattfindet ______________________________ (Name des Behandlers bzw. der Einrichtung) und erklären uns damit einverstanden, dass der Behandler / die Behandlerin gegenüber dem Studienpersonal Informationen bezüglich Begabung und Autismus-Symptomatik des Kindes weitergeben darf.

....
Name des Kindes in Druckbuchstaben

....	....
Ort, Datum	Name und Unterschrift des ersten **Sorgeberechtigten**
....	....
Ort, Datum	Name und Unterschrift des zweiten **Sorgeberechtigten**

Ich habe das Aufklärungsgespräch geführt und die Einwilligung des Teilnehmers oder der Teilnehmerin eingeholt.

....
Name des / der aufklärenden Studienmitarbeiter*in in Druckbuchstaben

....	....
Ort, Datum	Unterschrift des / der aufklärenden Studienmitarbeiter*in
