# Supplementary material for: Smartphone-based behaviour analysis for challenging behaviour in intellectual and developmental disabilities and autism spectrum disorder – Study protocol for the ProVIA trial
Source: Front Neurosci. 2022 Oct 13;16:984618. doi: 10.3389/fnins.2022.984618 (PMC9610118; doi:10.3389/fnins.2022.984618)
Supplement: Supplementary file 2 [file Table_1.DOCX]

**Supplementary Table 1.** Interventions for the main causal factors of challenging behaviour

| Our **living conditions** comprise the areas of self-determination, experience of competence, shaping of the environment and interpersonal relationships. People with ASD/IDD often live under unfavourable conditions, especially as caregivers want to protect them and take responsibility away from them. They often have little freedom of choice and are often not challenged according to their abilities. CB can arise from such unfavourable living conditions. In the app, caregivers are sensitised to the living conditions of the children, guided to better perceive the children's competences and to give them more freedom and responsibility for everyday decisions. |
| --- |
| Sometimes challenging behaviour occurs more frequently **when certain people are present**. Reasons for this can be, for example, special sensory stimuli (e.g. strong perfume) or characteristics (e.g. impatience, insecurity in dealing with the child) of the person. Often the child’s behaviour also has positive consequences for the child when interacting with this person. Thus the CB is reinforced. Via the app, caregivers are guided in observation and are given strategies to modify the personal factors that may contribute to the CB. |
| The **relationship between caregivers and children** with ASD/IE is often strained for several reasons. On the one hand, caregivers have to get used to the situation of having a child with certain limitations, and adapt their expectations regarding family life to the new circumstances. They have to keep themselves informed about ASD/IDD, and frequent visits to the doctor are often part of everyday life. Everyday life as a whole becomes more challenging. In this "problem-solving focus", many caregivers have hardly any positive experiences with the child. The child can sense when the caregiver has a negative attitude towards them as a result of the poor relationship. This in turn can lead to CB. The app presents interventions that promote attachment, e.g. by encouraging acceptance of the new reality, creating positive moments together, or fostering recognition of the child's positive qualities and reciprocity. |
| **Group situations** are a challenge for many people with ASD/IDD. In addition to the social skills required for successful interactions with other people, the unpredictability of social situations as well as sensory conditions are stressors that can lead to CB. The app guides caregivers to identify the individual stress-inducing factors regarding group situations that are relevant for the child. They receive recommendations to modify group situations in such a way that they create the least amount of stress for people with ASD/IDD. |
| **Transitions** (e.g. from playing to working, from one activity to the next, change of caregiver) are a challenge for people with ASD/IDD, because they often have difficulty orienting to the new circumstances. They slowly get used to the new situation and often feel a high level of tension, which can manifest in challenging behaviour. Caregivers receive psychoeducational information and concrete instructions on how to favourably shape and accompany transitional situations, i.e. via including visualisation aids. |
| Predictability is essential for people with ASD/IDD. They benefit greatly from knowing details regarding upcoming situation (e.g. who will be there, what will the environment look like, what is going to happen and what is expected of them). **New or altered situations** are often unsettling due to a lack of orientation or difficulty with processing emotions such as insecurity or anxiety. CB is often an expression of tension and helplessness. Via the app, caregivers are sensitised to spot new or changed situations from the child's point of view. By teaching techniques that help the caregivers to prepare for and structure new or altered situations in such a way that the child can more easily adapt to them, they gain competence in preventing high tension levels and thus CB. |
| CB does not occur randomly throughout the day, but is often linked to **specific situations that contain risk factors** which make them difficult for the child to handle. CB can be prevented with careful planning for those situations. Via the app, caregivers define specific risk aspects of the situations and learn to anticipate them. They identify helpful tools for managing the situation and define necessary preparatory steps. They learn to clearly define and communicate expectations for the child and rules pertaining to the situation as well as consequences of desired behaviour versus CB. Furthermore, they are encouraged to debrief after the situation and assess what strategies worked. Increasing caregivers’ confidence in their own competence and thus reducing their tension level in difficult situations creates favourable framework conditions for the child and can further reduce the likelihood for CB. |
| CB can be a sign of **excessive demands**. Children with ASD/IDD have various limitations that need to be taken into account when setting tasks, since they need additional support. The children often cannot express that they are overwhelmed or frustrated, and they often cannot ask for help. Usually, when the child shows CB, an adult comes and assists them or removes them from the situation. Therefore, CB is a successful problem solving strategy for the child. In the app, caregivers are guided to recognise individual signs of excessive demands for the child and to offer appropriate support. |
| CB often occurs after giving one or more **instructions** to the child. This may be due to deficits in attention, task comprehension or action planning, among other things. In addition to psychoeducational information sensitising caregivers to the child's difficulties they learn how to give effective instructions following the THOP approach [61]. In order to prevent unreasonable expectations, the chapter furthermore outlines that in the context of child development it is normal and healthy that children do not always directly follow all instructions given by caregivers. |
| Children with ASD/IDD have a high need for structure. A structured environment means: We know what we are supposed to do. And when, for how long, where and how we are supposed to do it. The more predictable a situation, the less stress a person experiences and the less likely they are to display CB. The app introduces methods for structuring space, time and activities following the TEACCH approach [59]. In addition to guidance on how to structure children's daily lives in general, caregivers learn appropriate strategies (e.g. using visual schedules, picture cards or timers) to make **unstructured situations** predictable and to support the child in coping with these situations. |
| When our **basic physical needs** (food, drink, sleep, health) are not adequately met over a long period of time, we become vulnerable. We are quicker to irritate and sometimes show exceedingly strong emotional reactions. We find things difficult that we could normally manage without difficulty. We are more easily overwhelmed and conflict situations can arise. This app explains the basic needs of children and presents strategies to ensure that these needs are met in order to prevent CB. |
| **Illness and pain** can give rise to CB. Due to deficits in communication skills and proprioception, people with ASD/IDD often cannot communicate to their caregivers that they are in pain or sick. When a child is in pain, it are more likely to feel stressed or overwhelmed and may not be able to regulate own emotions well. The app supports caregivers in recognizing signs of pain or physical or mental illness. Furthermore, they are provided with practical recommendations from everyday clinical practice (e.g. scheduling regular medical check-ups, especially dental). |
| Many people with ASD/IDD have **sensory processing difficulties**, i.e. hypersensitivity or hyposensitivity on one or more sensory channels. This altered sensory perception can cause stress and tension in situations where those stimuli are present, which in turn can manifest in CB. Caregivers are trained in recognising the child’s sensory difficulties with the help of a checklist. In case of hypersensitivity, appropriate strategies for attenuating sensory stimuli are presented for each sensory channel. For hyposensitivity, in addition to psychoeducational information depathologising sensory interests, users find suggestions for safely satisfying these sensory interests. |
| **Strong emotions and tension** play an important role in the development of challenging behaviour. A high level of stress or tension can result from both intrinsically pleasant emotions such as joy as well as from unpleasant emotions. Most people rate tension as an aversive state. Children with ASD/IDD are often unable to regulate this tension on their own. In this case, CB can be an expression of helplessness. Based on the concept of dialectical behaviour therapy [78], the app instructs caregivers to identify individual triggers of tension and to recognise signs of low, medium or high tension at an early stage. Strategies for prevention and regulation appropriate to the level of tension are taught. |
| **Behavioural consequences** are important for the emergence and maintenance of any behaviour. When CB has pleasant consequences, this signifies an unintentional reinforcement of the CB. The app teaches caregivers the basics of reinforcement learning. They learn to identify positive and negative consequences of the child's CB and to change the behavioural contingencies in a way that only desirable behaviour is reinforced. Reward schedules are introduced as tools for modifying behaviour though consequences. Caregivers can furthermore access information regarding parenting strategies, e.g. on how to effectively and appropriately express praise and criticism. |
