# Supplementary material for: Smartphone-based behaviour analysis for challenging behaviour in intellectual and developmental disabilities and autism spectrum disorder – Study protocol for the ProVIA trial
Source: Front Neurosci. 2022 Oct 13;16:984618. doi: 10.3389/fnins.2022.984618 (PMC9610118; doi:10.3389/fnins.2022.984618)
Supplement: Supplementary file 3 [file Table_2.DOCX]

**Supplementary Table 2.** Interventions for at strengthening caregivers’ resources

| Taking care of other people requires a lot of time and energy. One's own needs are often put on the back burner. Caregivers have a significantly increased risk of becoming mentally ill themselves. Therefore, **practicing self-care** is an important topic. It is the basis for being an effective helper to others in the long run. With the help of materials such as a self-care diary, the app supports caregivers in integrating self-care into everyday life in order to increase resilience and prevent burn-out. In addition to psychoeducational information on the crucial importance of self-care, users find exercises such as:   - Planning enjoyable activities (with list of enjoyable activities and goal planner) - Practising mindfulness and relaxation (with various exercises, some in an audio format) - Building resources (e.g., The Benevolent Companion, My Self-Esteem House, The Inner Critic, My Inner Place of Well-Being, Self-Compassion) - Tending to your own basic needs - Communicating needs and asking for support |
| --- |
| Caregivers face many challenges in everyday life. Functional **coping strategies for stress** can have a de-escalating effect in critical situations and contribute to maintaining their mental health in the long term. The app helps caregivers identify individual stressors, showcases preventative exercises (e.g., integrating mindfulness and relaxation into everyday life, setting priorities, problem-solving training, time management skills). In addition, they are introduced to the modification of dysfunctional thinking as a cognitive intervention. |
| **Staying calm under pressure** is an important issue many caregivers face. CB behaviour can push carers to their physical and psychological limits. Feeling tense or stressed is normal under those circumstances. In order to enable caregivers to stay calm especially in critical situations, the DBT-based chapter helps identify early signs of stress and introduces skills to reduce tension (e.g. body-based skills, cognitive skills, skills using imagination). |
| **Dealing with emotions in a healthy way** is material for caregivers’ well-being. When dealing with children with ASD/IDD, caregivers experience not only positive but also negative emotions, such as shame about the child's behaviour in public or anger due to the belief that the child can control its behaviour and intentionally ‘provokes’ others. Experiencing these feelings is often stressful for the caregivers. The app provides psychoeducational information regarding the purpose and the importance of negative emotions. Caregivers can then learn strategies for reducing their susceptibility to strong emotions and for modifying negative emotions in cases where their quality or intensity is inappropriate to the situation. |
| Caring for children with ASD/IDD is a challenging task. Even professional carers know the feeling of being overwhelmed. Often, staff members of residential and care facilities, for example, reach their physical and mental limits. This chapter contains a selection of contents that can be helpful for supporting **teams** and team members. |
